# Urinary Proteomics Reveals Promising Biomarkers in Menstrually Related and Post-Menopause Migraine

**DOI:** 10.3390/jcm10091854

**Published:** 2021-04-24

**Authors:** Elisa Bellei, Stefania Bergamini, Cecilia Rustichelli, Emanuela Monari, Michele Dal Porto, Alessandro Fiorini, Aldo Tomasi, Anna Ferrari

**Affiliations:** 1 Department of Surgery, Medicine, Dentistry and Morphological Sciences with Transplant Surgery, Oncology and Regenerative Medicine Relevance, Proteomic Lab, University of Modena and Reggio Emilia, Via del Pozzo, 71, 41124 Modena, Italy; stefania.bergamini@unimore.it (S.B.); emanuela.monari@unimore.it (E.M.); aldo.tomasi@unimore.it (A.T.); 2 Department of Life Sciences, University of Modena and Reggio Emilia, Via G. Campi 103, 41125 Modena, Italy; cecilia.rustichelli@unimore.it; 3 School of Pharmacology and Clinical Toxicology, University of Modena and Reggio Emilia, Via del Pozzo, 71, 41124 Modena, Italy; dalportomichele@gmail.com (M.D.P.); alfiorx@yahoo.it (A.F.); 4 Unit of Medical Toxicology, Headache Centre and Drug Abuse, Department of Biomedical, Metabolic and Neural Sciences, University of Modena and Reggio Emilia, Via del Pozzo, 71, 41124 Modena, Italy; anna.ferrari@unimore.it

**Keywords:** proteomics, urinary proteins, LC-MS/MS, menstrually related migraine, post-menopause migraine, headache, migraine

## Abstract

Migraine is an invalidating neuro-vascular disorder largely spread in the world population. Currently, its pathophysiology is not yet completely understood. The purpose of this study was to investigate the urinary proteome of women suffering from menstrually related migraine (MM) and post-menopause migraine (PM) in comparison with non-headache women as controls, to search potential biomarkers of these migraine sub-types. Urine samples were analyzed by mono-dimensional gel electrophoresis (SDS-PAGE) and two-dimensional gel electrophoresis (2DE) coupled to liquid chromatography-mass spectrometry (LC-MS/MS). Twenty-one urinary proteins were found significantly dysregulated in MM and PM (*p* < 0.05). The STRING Analysis database revealed interaction between 15 proteins, which were mainly involved in the immune and inflammatory response. Seven of the most considerable proteins were further quantified by western blot: protein S100A8 (S10A8), up-regulated in MM, uromodulin (UROM), alpha-1-microglobulin (AMBP), gelsolin (GELS), prostaglandin-H2 D-isomerase (PTGDS), over-expressed in PM, apolipoprotein A-I (APOA1), and transthyretin (TTHY), respectively down- and up-regulated in both migraineur groups vs controls. These candidate biomarkers might be involved in the neurophysiological network of MM and PM, thus helping to better understand the pathophysiology of these migraine forms. If validated in large-scale studies, this protein cluster could become a distinctive target for clinical applications in migraine diagnosis and treatment.

## 1. Introduction

Menstrually related migraine (MM) is a subclass of migraine that affects 42–61% of women with migraine disease [1]. Menstrual attacks are often much more disabling, of longer duration, and more resistant to treatment than migraine attacks occurring outside the perimenstrual period [2,3]. For millions of women around the world, menstruation regularly and often catastrophically disrupts their physical, mental, and social wellness. Additionally, women suffering from MM have to worry about how to manage the severe attack usually associated with menstruation [1,4]. The menopause is a physiological period in a woman’s life corresponding with the end of fertility. The decrease in estrogen levels can generate various disorders and symptoms, both of an autonomic nature (hot flashes, profuse sweating, palpitations, blood pressure alterations, and sleep disturbances) and of a psycho-affective nature (anxiety, irritability, mood changes, and impaired concentration and memory), which cause significant discomfort [5,6]. In population studies, the prevalence of post-menopause migraine (PM) drops by about half, due to hormonal stability [7,8]. On the contrary, studies carried out among patients who refer to specialized headache centers reported no improvement or even a worsening of migraine after menopause [6]. Therefore, both MM and PM are highly disabling conditions within the spectrum of migraine disorders which affect several women. Nevertheless, women’s health concerns are generally underrepresented in basic and translational research. In particular, migraine is often underreported, despite being associated with significant disability [7]. At present, no specific preventive treatments have been approved for MM and PM, as their pathogenesis is not yet completely explained [4,8]. It would, therefore, be essential to gain a deeper understanding of the biological characteristics of these types of migraine, to alleviate suffering and improve the quality of life of females living with migraine [1]. Currently, proteomics is one the most significant methodology for the identification of the overall protein content of cells, tissues, or biofluids [9]. Together with other “*omics*” technologies, especially genomics and metabolomics, proteomics, and techniques derived therefrom (miniaturized proteomics) are involved in different research settings, such as detection of diagnostic biomarkers, alteration of expression patterns in response to different signals, and interpretation of functional protein pathways in various diseases to understand their pathogenetic mechanisms [10]. The “*omics*” synergism recognizes migraine as an ideal research model due to its multifactorial nature [11]. However, at present, few proteomics studies have been developed in the attempt to identify promising biomarkers and new physiopathological pathways associated with migraine and other types of headache disorders. In the last few years, our group widely investigated the medication-overuse headache (MOH) by proteomics, to discover candidate urinary biomarkers of drug-induced nephrotoxicity [12,13] and allodynia [14], as well as serum targets of chronic pain in MOH patients [15,16] and in an animal model of chronic neuropathic pain [17]. Moreover, we have examined the serum proteome of female suffering from MM and PM, in the search of potential serum biomarkers [18]. A summary table showing the proteomic studies conducted up to now in the field of migraine disorder and reported in this paper can be viewed in Supplementary Table S1.

The aim of the present work is to reinforce and expand the previous study [18], now focusing on the urinary proteome. Indeed, urine sample offers some benefits in clinical proteomics, since it is readily available in large quantity, and its collection is easy and non-invasive [19]. We employed both mono-dimensional gel electrophoresis (SDS-PAGE) and two-dimensional gel electrophoresis (2DE) coupled to liquid chromatography-mass spectrometry (LC-MS/MS) to analyze the urinary proteomic profile of MM and PM women, in comparison with non-headache women as control group. The differentially expressed proteins were then subjected to protein-protein network assessment by STRING Analysis database and some of the most significant proteins were further validated and quantified by western blot.

## 2. Materials and Methods

### 2.1. Subjects and Procedures

The study involved three different groups: (1) MM group, composed of 15 women aged between 18 and 45 years (mean age ± SD: 34.8 ± 7.1) diagnosed with menstrually related migraine without aura according to the diagnostic criteria of the International Classification of Headache Disorders, 3rd edition (ICHD-3, appendix A1.1.2) [20], (2) PM group, which comprised 15 women, aged no more than 65 years (mean age ± SD: 57.0 ± 5.0) in menopause (spontaneous amenorrhea) for at least 12 months, not induced by a medical condition, who had suffered from migraine without aura and MM [20] during their fertile age, and continued to be affected by migraine without aura [20] in post-menopause, and (3) CTRL group, composed of 15 non-headache women, aged between 18 and 45 years (mean age ± SD: 29.2 ± 7.2) as control, age-matched with MM group. Length of migraine history (years ± SD) was 16.8 ± 8.5 in MM group and 33.1 ± 13.5 in PM group; frequency of migraine (days/3 months ± SD) was 25.7 ± 17.2 and 27.3 ± 19.2 in MM and PM groups, respectively. Only subjects without major medical or psychiatric comorbidities and with normal kidney and liver functions were included in the study. Women taking hormonal therapy (contraceptive or post-menopause therapy) and migraine prophylaxis were excluded. The three groups of women did not differ in lifestyle habits and comorbidities (one-way ANOVA test). Further details about the studied groups are described in our previous work [18] and are reported in Supplementary Table S2. The women with migraines were enrolled from consecutive patients attending, for the first time, the Headache Centre of the University Hospital of Modena; non-headache women were patients’ acquaintances. All women gave their written informed consent for inclusion before they participated in the study, which was conducted in accordance with the ethical principles of the Helsinki Declaration, last edition 2013, and the protocol was approved by the Ethics Committee of the Province of Modena, Italy (protocol n. 0013510/18).

### 2.2. Sample Collection and Pre-Analytical Treatment

In women of fertile age, urine for proteomic analysis was taken between the seventh and tenth day, starting from the first day of menstruation and, in migraine women (MM and PM groups), at least two days after the last migraine attack. Urine samples (morning midstream) were collected into sterile containers reaching a final volume of about 20–30 mL, and immediately placed on ice for the transport to the lab. Each sample was centrifuged at 800× *g* for 10 min at 4 °C to remove cell debris and contaminants. Then, samples were desalted and the urinary proteins concentrated by means of specific filter device, 3 kDa molecular weight cut-off (Amicon Ultra, Millipore, Burlington, MA, USA). In this way, the samples were approximately 50 times more concentrated than the original ones. Total protein content was measured spectrophotometrically at λ = 595 nm, using the protein Assay Dye Reagent (Bio-Rad Laboratories, Hercules, CA, USA) and bovine serum albumin (Sigma, St. Louis, MI, USA) as standard for the calibration curve.

### 2.3. Mono-Dimensional Gel Electrophoresis (SDS-PAGE)

SDS-PAGE was performed under reducing conditions according to the Laemmli’s method, as previously described [16]. Pooled urinary samples (5 pools *per* group) were diluted 1:1 with the Laemmli sample buffer added of 20% 2-mercaptoethanol (Merck KGaA, Darmstadt, Germany) and heated at 95 °C for 5 min. Samples (20 mL/well) were then loaded onto 4–12% precast gradient gel (Novex NuPAGE^TM^, Thermo Fisher Scientific, Waltham, MA, USA) and the electrophoretic run was carried out in a mini-gel apparatus (MiniPROTEAN vertical cell, Bio-Rad Laboratories) using MES 1X running buffer (Life Technologies Italia, MB, Italy). Urinary protein bands were finally stained with Coomassie Blue G-250 (Sigma) and gel images were acquired by a calibrated densitometer (model GS-800, Bio-Rad Laboratories). The QuantityOne 1-D image analysis software, version 4.6.7 (Bio-Rad Laboratories) was used to detect a differential protein expression among the groups, according to the staining intensity and bands volume.

### 2.4. Two-Dimensional Gel Electrophoresis (2DE)

Urinary proteins were separated and analyzed by 2DE. The first-dimension separation was conducted by isoelectrofocusing (IEF) in a PROTEAN IEF^®^ cell (Bio-Rad Laboratories), mixing 80 μg of proteins from each pool with the lysis buffer (6 M urea, 2 M thiourea, 4% CHAPS, 25 mM DTT, 0.2% ampholytes, all from Bio-Rad Laboratories) to a final volume of 300 μL/sample. The solution was then loaded onto 17-cm immobilized pH gradient (IPG) strips, pH range 3–10 (Ready Strip^TM^, Bio-Rad Laboratories), and analyzed as previously reported in detail [18]. The second-dimension separation was performed in a PROTEAN^®^ II xi cell vertical system (Bio-Rad Laboratories), connected to a refrigerated bath circulator set at constant 10 °C (Cryostatic bath, MPM Instruments S.r.l., MB, Italy). Large size 8–16% polyacrylamide gradient gels (29:1 acrylamide/bis solution, 1.5 M Tris, pH 8.8, 10% SDS, 1% TEMED, 10% ammonium persulfate, from Bio-Rad Laboratories) and TGS 1X running buffer (Bio-Rad Laboratories) were employed for the electrophoretic run. Gels were subsequently incubated overnight at room temperature in a fixing buffer solution (30% ethanol/10% acetic acid, Carlo Erba, Milan, Italy) and then sensitized in the enhancer solution (0.5 M potassium acetate, 0.3% potassium tetrathionate, 30% ethanol, from Merck) before staining with 0.2% silver nitrate (Sigma) for 1 h in the dark. Finally, protein spots were developed by a solution composed of 3% potassium carbonate, 0.03% sodium thiosulfate (Merck), and formaldehyde (Sigma-Aldrich, St. Louis, MI, USA). All reagents and solvents were of analytical grade. 

Each gel image was acquired by a calibrated densitometer and exported to the PDQuest 2D image analysis software program, version 7.3.1 (Bio-Rad), to accurately detect the significantly increased or decreased protein spots among the studied groups, based on spot stain intensity and area. The differentially expressed spots were cut from the corresponding gel and stored at −20 °C until their processing for MS analysis.

### 2.5. LC-MS/MS Analysis

The selected protein spots excised from the gels were subjected to an “in-gel” protein spot digestion protocol, as previously described [12]. Briefly, the spots were first de-stained by a dark incubation (1:1 *v*/*v*, 30 mM potassium hexacyano-ferrate(III)/100 mM sodium thiosulfate solution, Sigma-Aldrich), after which the included proteins were reduced and alkylated with 10 mM DTT (Bio-Rad Laboratories) and 55 mM iodoacetamide (Bio-Rad Laboratories), respectively. After drying, spots were covered with a trypsin solution (Promega, Madison, WI, USA) and incubated at 37 °C. Extracted peptides were analyzed using an UHPLC-MS QExactive™ (Thermo Scientific) system, composed of UHPLC 3000 Ultimate System coupled to an ESI-QExactive™ Hybrid Quadrupole-Orbitrap™ mass spectrometer (LC-ESI-QO-MS/MS System), as previously fully described [18]. Before MS analysis, dried samples were resuspended in water/acetonitrile/formic acid (95:3:2, *v*/*v*), sonicated for 10 min at room temperature, and centrifuged at 12,100× *g* for 10 min. Separations were carried out in gradient mode on a ZORBAX RRHD Eclipse Plus C18 column (50 × 2.1 mm ID.; 1.8 μm particle size; Agilent, Santa Clara, CA, USA) with a mobile phase composed of 0.1% aqueous formic acid solution and acetonitrile. The analyses were controlled by Xcalibur™ software, (version 29 build 2926) and the raw data were converted into mascot generic format using MsConvert (version 3.0.10730, ProteoWizard tools, Palo Alto, CA, USA). The data were analyzed by MASCOT search engine (version 2.4, Matrix Science, Boston, MA, USA) against the databases UniProt, for peptide sequences, and C-RAP, for contaminants. The search parameters were set as follows: trypsin as proteolytic enzyme, carbamidomethyl-cysteine as fixed modification, deamidated (NQ) and oxidated (M) methionine as variable modifications, one missed trypsin cleavage allowed, and mass tolerance set at 10 ppm for the precursor ions and 0.05 Da for the product ions. An automatic decoy database search was used to estimate the false discovery rate, which was adjusted to <1%.

The identified proteins were then subjected to protein-protein interaction network analysis by STRING Analysis database, version 11.0 (https://string-db.org (accessed on 28 December 2020)). Afterward, some potential biomarkers were selected for further validation and quantification by immunoblot.

### 2.6. Western Blot Analysis

Urinary proteins were separated on precast gel Bolt^TM^ 12% polyacrylamide Bis-Tris Plus (Life Technologies Italia, MB, Italy) and blotted onto nitrocellulose membranes, previously blocked with 5% non-fat milk solution. The membranes were incubated overnight at 4 °C with the following antibodies: protein S100-A8 (S10A8) rabbit polyclonal, 1:1000 dilution (Thermo Scientific), uromodulin (UROM) rabbit polyclonal, 1:500 dilution (Abcam, Cambridge, UK), alpha-1-microglobulin (AMBP) rabbit monoclonal, 1:1000 dilution (Abcam), gelsolin (GELS) rabbit polyclonal, 1:500 dilution (Thermo Scientific), prostaglandin-H2 D-isomerase (PTGDS) rabbit polyclonal, 1:500 dilution (Abcam), apolipoprotein A1 (APOA1) rabbit polyclonal, 1:500 dilution (Abcam), and transthyretin (TTHY) rabbit monoclonal, 1:1000 dilution (Abcam). Membranes were then incubated for 1 h with the secondary antibody (Anti-Rabbit IgG VHH Single Domain Antibody HRP-conjugated, 1:6000 dilution, Abcam). Protein signals were developed using the ECL technique (WesternSureTM PREMIUM Chemiluminescent substrate, LI-COR Biosciences, Lincoln, NE, USA) and detected by the C-DiGit^®^ Blot Scanner (LI-COR Biosciences). The Image Studio™ Lite software (LI-COR Biosciences, Lincoln, NE, USA) was finally used for signals acquisition and quantification (represented by arbitrary units, AU). Human serum samples were employed as positive or negative controls.

### 2.7. Statistics

The one-way ANOVA test was used to compare the characteristics of the three groups of women, considering a *p*-value < 0.05 as statistically significant. A fold-change in the protein expression level of at least 1.5 between migraineurs and non-headache control women, detected by QuantityOne and PDQuest image analysis software, was considered a significant difference among the groups. Data obtained by SDS-PAGE, 2DE and immunoblot analysis were compared and statistically evaluated by the Student’s *t*-test: statistical significance was defined as *p* < 0.05. All data were expressed as mean ± standard deviation (SD).

## 3. Results

### 3.1. SDS-PAGE and LC-MS/MS Analysis

**Figure 1:**
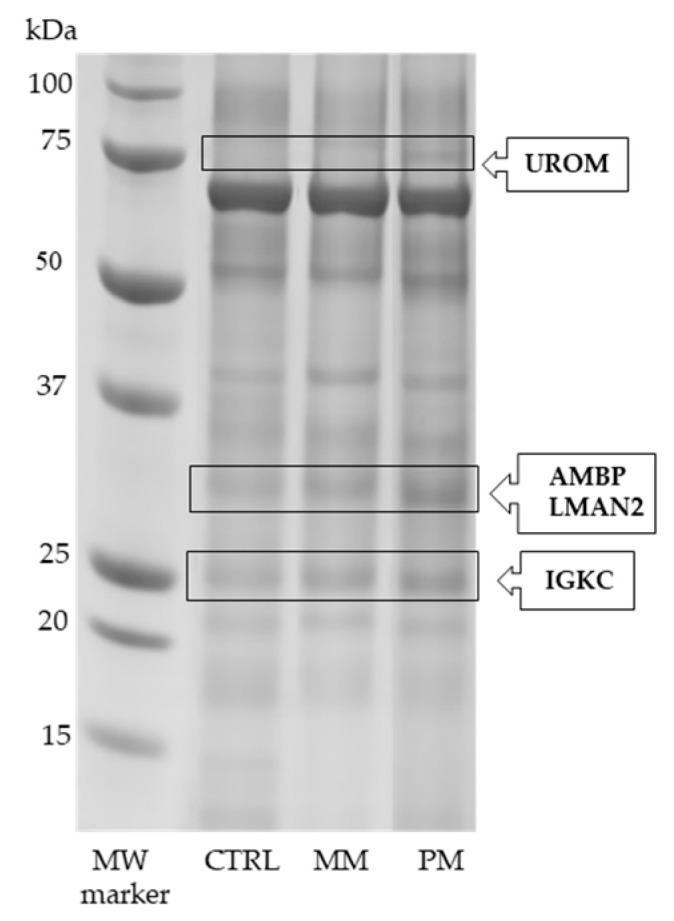
SDS-PAGE urinary protein profile. The differentially expressed bands among the three groups are bordered by rectangles, alongside the abbreviated protein names: UROM (uromodulin), AMBP (alpha-1-microglobulin), LMAN2 (vesicular integral-membrane protein VIP36), IGKC (immunoglobulin kappa constant). First lane: molecular weight (MW) marker, expressed as kDa (PrecisionPlus Protein Standards, Bio-Rad); CTRL, control group; MM, menstrually related migraine group; PM, post-menopause migraine group.

The analysis of the urinary proteome by SDS-PAGE and QuantityOne 1-D image software revealed some protein bands differentially expressed among the considered groups (Figure 1). Proteins were identified by LC-ESI-QO-MS/MS analysis, as reported in Table 1. Uromodulin (UROM) showed a significantly up-regulation in PM group vs both the CTRL (fold-change: +2.00, *p* = 0.013) and MM group (fold-change: +1.95, *p* = 0.011). Alpha-1-microglobulin (AMBP) and vesicular integral-membrane protein VIP36 (LMAN2) were both increased in PM group compared to CTRL and MM groups (fold-change: +2.51, *p* = 0.001 and +1.67, *p* = 0.005, respectively). Finally, the immunoglobulin kappa constant (IGKC) was significantly increased (*p* = 0.013) only in PM vs CTRL (fold-change: +1.58).

### 3.2. DE and LC-MS/MS Identification

**Figure 2:**
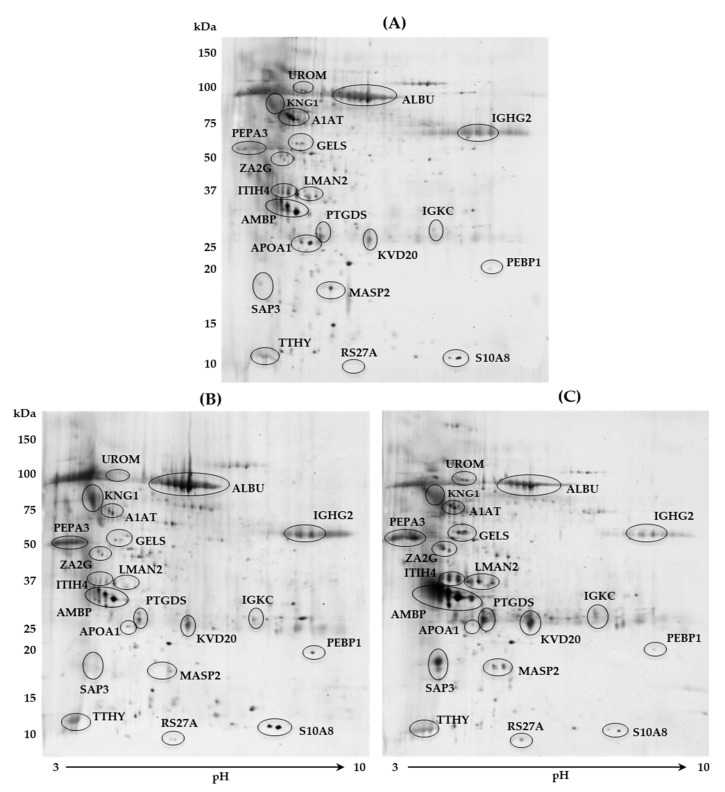
Representative 2DE urinary maps. The differentially expressed protein spots among (**A**) control group, (**B**) menstrually related migraine group, and (**C**) post-menopause migraine group are evidenced by ellipses; protein entry names refer to those reported in Table 2. Two-dimensional separation was performed by 17 cm IPG strip, pH range 3–10 (first-dimension), and 8–16% polyacrylamide gradient gel (second-dimension). Molecular weight marker (PrecisionPlus All Blue, Bio-Rad) is expressed in kilodalton (kDa).

Twenty-one protein spots were detected as differentially expressed (*p* < 0.05) among the three groups by 2DE (Figure 2) and PDQuest analysis software. Proteins were subsequently identified by LC-MS/MS analysis (Table 2). Out of these proteins, five were significantly increased only in the MM group (Figure 2B) vs. CTRL group (Figure 2A) and PM group (Figure 2C), comprising protein S100-A8 (S10A8), kininogen-1 (KNG1), albumin (ALBU), immunoglobulin heavy constant gamma 2 (IGHG2), and phosphatidylethanolamine-binding protein 1 (PEBP1). Otherwise, 12 proteins were found to be increased only in the PM group vs CTRL and MM groups: UROM, AMBP, LMAN2, IGKC, inter-alpha-trypsin inhibitor heavy chain H4 (ITIH4), zinc-alpha-2-glycoprotein (ZA2G), mannan-binding lectin serine protease 2 (MASP2), gelsolin (GELS), prostaglandin-H2 D-isomerase (PTGDS), ganglioside GM2 activator (SAP3), immunoglobulin kappa variable 3D-20 (KVD20), and ubiquitin-40S ribosomal protein S27a (RS27A). Regarding UROM, AMBP, and LMAN2, the 2DE findings were fully consistent with those previously obtained by SDS-PAGE (Figure 1 and Table 1), whereas IGKC expression was comparable only in PM vs control women. Finally, two proteins were down-regulated in both groups of patients with migraine vs CTRL (Figure 2A), including apolipoprotein A-I (APOA1) and alpha-1-antitrypsin (A1AT), while two protein spots, identified as transthyretin (TTHY) and pepsin A-3 (PEPA3), were up-regulated in the same comparison.

In Table 2 are listed all the identified proteins with their entry name, primary full name, accession number, gene name (from the UniProt database), mass data, namely summary score, significant peptides, and significant sequences, and emPAI, the change in expression among the three groups (up- or down-regulation) and the main biological/molecular functions.

### 3.3. Protein Interactions Network by STRING

**Figure 3:**
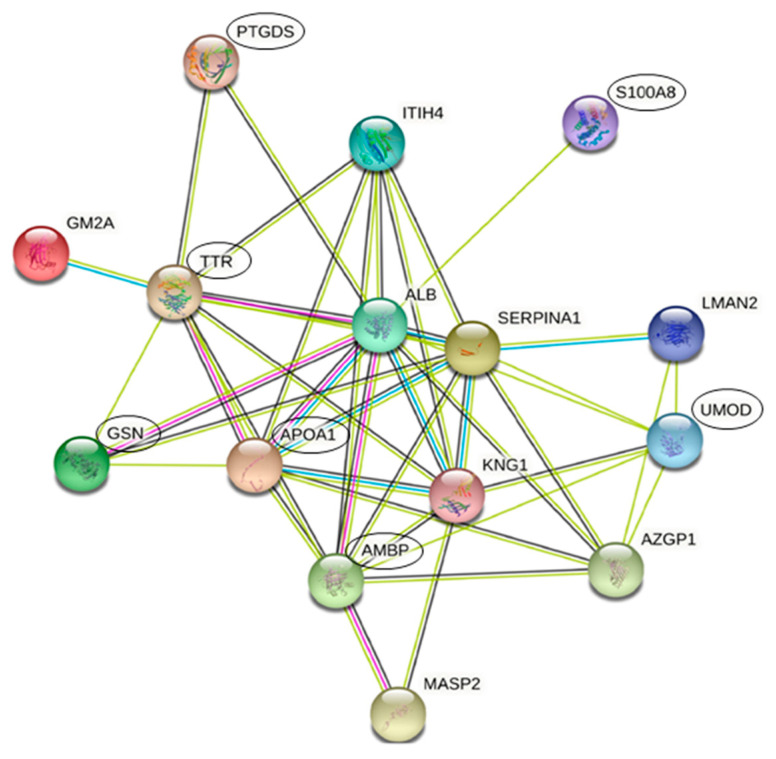
Functional protein-protein interactions by STRING. The pathways are shown as nodes (proteins, *n* = 15) and lines (protein-protein associations, *n* = 43). Proteins are shown with their gene names: **PTGDS**, prostaglandin-H2 D-isomerase; **ITIH4**, inter-alpha-trypsin inhibitor heavy chain H4; **S100A8**, protein S100-A8 (S10A8); **GM2A**, ganglioside GM2 activator (SAP3); **TTR**, transthyretin (TTHY); **ALB**, albumin (ALBU); **SERPINA1**, alpha-1-antitrypsin (A1AT); **LMAN2**, vesicular integral-membrane protein VIP36; **GSN**, gelsolin (GELS); **APOA1**, apolipoprotein A-I; **KNG1**, kininogen-1; **UMOD**, uromodulin (UROM); **AMBP**, alpha-1-microglobulin; **AZGP1**, zinc-alpha-2-glycoprotein (ZA2G); **MASP2**, mannan-binding lectin serine protease 2.

Protein-protein associations were evaluated using STRING Analysis database. As illustrated in Figure 3, the pathway map revealed the relationship (showed by 43 colored lines) between 15 proteins, reported with the name of the respective gene and indicated by nodes (average node degree = 5.73, average local clustering coefficient = 0.809, enrichment *p*-value < 1.0 × 10^−16^). Blue and pink lines indicate direct known interactions (from curated databases or experimentally determined); predicted interactions are indicated by green lines (gene neighborhood) and black lines (co-expression). Proteins enclosed in ellipses were further validated and quantified by western blot analysis. 

### 3.4. Western Blot Analysis

**Figure 4:**
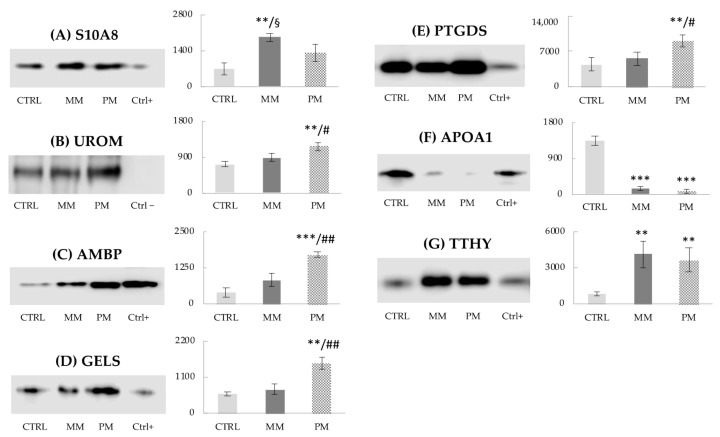
Protein signal intensity detected by western blot analysis: (**A**), signal expression of S10A8 (protein S100A8), increased only in MM vs. CTRL and PM groups; (**B**), expression of UROM (uromodulin); (**C**), AMBP (alpha-1-microglobulin); (**D**), GELS (gelsolin); (**E**), PTGDS (prostaglandin-H2 D-isomerase), up-regulated only in PM vs. CTRL and MM groups; (**F**), signal expression of APOA1 (apolipoprotein A-1), decreased in both MM and PM groups vs. CTRL group; (**G**), expression intensity of TTHY (transthyretin), up-regulated in both migraineurs groups vs. CTRL group. Serum sample was used as positive or negative control (Ctrl+/−). Protein signal intensity was expressed as arbitrary units in control group (CTRL), menstrually related migraine group (MM), and post-menopause migraine group (PM). Student’s *t*-test vs. CTRL group: ** *p* < 0.01, *** *p* < 0.001; vs. PM group ^§^ *p* < 0.05; vs. MM group # *p* < 0.05, ## *p* < 0.01.

Based on their significant biological functions, seven differentially expressed proteins, namely S10A8, UROM, AMBP, GELS, PTGDS, APOA1, and TTHY, were further validated and quantified by immunoblot (Figure 4). The analysis demonstrated protein expressions consistent with the results obtained by 2DE. In fact, S10A8 protein signal (Figure 4A) was found significantly higher in MM group with respect to CTRL (*p* < 0.01) and PM (*p* < 0.05). As additional validations, four proteins showed significantly more intense signal expression in PM group vs CTRL and MM groups, respectively: UROM (Figure 4B, *p* < 0.01; *p* < 0.05), AMBP (Figure 4C, *p* < 0.001; *p* < 0.01), GELS (Figure 4D, *p* < 0.01; *p* < 0.01), and PTGDS (Figure 4E, *p* < 0.01; *p* < 0.05). Finally, the expression intensity of APOA1 (Figure 4F) was confirmed to be strongly down-regulated (*p* < 0.001) in both migraineur groups compared to non-headache control women, and TTHY protein signals (Figure 4G) were up-regulated (*p* < 0.01) in the same comparisons, validating all previous results. Row data are available in the Supplementary Materials (Table S3). Human serum was used as experimental control, since these proteins, present in blood, are filtered by the kidney and excreted in urine (positive control). Only UROM is not present in serum, because it is exclusively produced in the kidney and secreted in urine via proteolytic cleavage (Figure 4B, serum signal absent; serum = negative control). Protein quantitative representation (expressed in arbitrary units) is provided by the histograms reported in Figure 4, alongside each relative western blot image.

## 4. Discussion

Migraine is the most common neurological disorder and the second most disabling human condition, of which the pathogenesis is favored by a combination of genetic, epigenetic, and environmental factors. Nowadays, the detection of useful migraine biomarkers is still a challenge, reflecting the disease complexity [21].

In this proteomic study, we discovered 21 urinary proteins with a significantly different expression among MM, PM, and CTRL groups (Figure 2 and Table 2). Of these proteins, 15 were closely connected by STRING analysis (Figure 3): S10A8, KNG1, ALBU (up-regulated in MM group), ITIH4, UROM, AMBP, LMAN2, ZA2G, MASP2, GELS, PTGDS, SAP3 (up-regulated in PM group), APOA1 along with A1AT, and TTHY (decreased and increased, respectively, in both migraineur groups vs. CTRL).

S10A8 is a calcium-binding protein that belongs to the S100 family, abundantly expressed in neutrophils and macrophages and significantly increased in almost all types of inflammation [22]. Together with S100A9, it can form a stable heterodimer that is actively released during inflammation. The S10A8/A9 complex also exhibits anti-inflammatory properties, modulating the production of pro-inflammatory mediators such as cytokines, chemokines, and nitric oxide, to avoid tissue damage caused by overwhelming inflammation [23]. S10A8 was validated and quantified by western blot analysis (Figure 4A); its increase was previously also observed in urine of MOH patients [13], thus supporting the role of inflammation and the activation of anti-inflammatory responses in migraine. KNG1 is the precursor protein for the plasma kallikrein-kinin system, which has pro-inflammatory, prothrombotic, and vasoactive properties [24]. This system is involved in inflammatory-like responses during the normal functions of the ovary and uterus, which seem to be regulated by estrogen [25]. Increased KNG1 levels were also detected in serum of MOH patients vs. non-headache controls [16]. Therefore, these results contribute to enhance the hypothesis about a reasonable activation of the inflammatory system in MM. Moreover, other studies showed altered urinary levels of KNG1 in endometriosis [26], and of ALBU e ITIH4 in ovarian carcinoma [27]. ITIH4 is an acute-phase inflammatory protein belonging to a super-family of protease inhibitors. It was earlier also found up-regulated in urine of MOH patients [13] and in serum of MM women [18]. In our present study, we found an over-expression of KNG1 and ALBU only in the MM group, and of ITIH4 in the PM group. The dysregulation of these proteins could derive from uterine and/or ovarian tissue modifications, which may occur during the menstrual cycle and in the post-menopause period. Indeed, the menopause is a crucial stage in women’s lives, leading to several physiological changes, especially hormonal variations [5,6].

Other proteins that were significantly increased in PM group vs. both CTRL and MM groups were UROM, AMBP, and LMAN2. These proteins were first identified by SDS-PAGE and LC-MS/MS (Figure 1 and Table 1) and then confirmed by 2DE and LC-MS/MS analysis (Figure 2 and Table 2). UROM is exclusively produced by the kidney and is the most abundant protein excreted in the normal urine by proteolytic cleavage. Its physiological role is not yet fully recognized, but it may serve as a receptor for binding and endocytosis of some cytokines (IL-1, IL-2) and TNF [28]. AMBP, or alpha-1-microglobulin, has an immunomodulatory function and inhibitory activities. Its expression is regulated by the same pro-inflammatory stimuli that trigger the acute phase response and the synthesis of C-reactive protein, serum amyloid alpha, and fibrinogen, all considered as markers of vascular inflammation and atherosclerosis. Evidence suggests that the urinary excretion of AMBP reflects the overall inflammatory status in patients with arterial hypertension and normal renal function [29]. Both UROM and AMBP were further demonstrated to be increased by western blot analysis (Figure 4B and Figure 4C, respectively). Moreover, they were previously found over-expressed in urine [12,13,14] and serum [16] of MOH patients and MM and PM women [18]. Based on their involvement in immune and inflammatory processes, these proteins found to be increased in PM women may be hypothetical biomarkers of the migraine permanence even after menopause. LMAN2 plays a role as an intracellular lectin in the early secretory pathway. A recent study reported the dysregulation of LMAN2 gene, through DNA methylation changes, in multiple system atrophy, which could be shared with others neurodegenerative diseases [30]. Additional proteins found to be increased in PM women were: ZA2G, MASP2, GELS, PTGDS, and SAP3. ZA2G is multidisciplinary polypeptide of which the expression is regulated by glucocorticoids. Its structural organization and fold are similar to MHC class I antigen-presenting molecule, so it may play a role in immune response [31]. ZA2G is also implicated in the regulation of adipose tissue metabolism in overweight/obese postmenopausal women due to inhibition of key enzymes in the lipogenesis pathway [32]. MASP2 is a specific mannose-binding lectin-associated (MBL) serine protease with different functions, such as complement cascade activation, mediation of innate immune defense against infections, recognition of altered self-structures, and modulation of inflammation [33]. Inflammatory responses may be altered after menopause and predispose to cardiovascular disease (CVD). It has been reported that MBL gene polymorphism is associated with lower risk factors for CVD in postmenopausal women [34]. Furthermore, in an experimental model of stroke, MASP2-deficient mice showed significantly reduced neurological deficits and histopathological damage after transient ischemia and reperfusion compared to wild-type or control-treated mice [35]. According to these findings, MASP2 might be used as an earlier biomarker for CVD risk in PM women, contributing to protect against CVD development in this population. Interestingly, as described for MASP2, GELS (together with estradiol, E2) has also been reported as a marker for the detection and diagnosis of CVD after menopause [36]. GELS is an actin-binding protein involved in a variety of both physiological and pathological processes, such as apoptosis, signal transduction, transcriptional regulation, and modulation of the inflammatory and immune response. Changes in blood concentrations of GELS, and consequently, in its urinary excretion, can result from inflammatory reactions induced by CNS infections or from non-specific inflammatory responses and actin release from axonal damage [37]. Considering that persistent neuroinflammation in the CNS is accompanied with the pathological development of neurodegenerative diseases (including multiple sclerosis, Alzheimer’s disease, encephalitis), the elevated level of urinary GELS found in PM group (Figure 4D) may represent a prospective target for neurological diseases. Likewise, PTGDS was significantly elevated in PM (Figure 4E). This is a prostaglandin synthase that we already found to be over-expressed in urine of MOH patients [13,14,15] and in rats with neuropathic pain [17], confirming its alteration in headache and other pain conditions. The role of falling in estrogen levels is believed to increase the susceptibility of blood vessels to prostaglandins, which have been implicated in neurogenic inflammation [38]. Lastly, SAP3 is a non-enzymatic essential cofactor exhibiting lipid transport and cholesterol transfer activities (UniProt database); accordingly, it could take part in the alterations of the plasmatic lipidome reported in postmenopausal women [39]. Therefore, these results indicated an increase expression, in PM women, of proteins involved in inflammatory response and lipid metabolism modification, which may lead to the development of metabolic syndromes, including CVD and type-2-diabetes [40].

Only two proteins were down-regulated in both MM and PM groups compared to CTRL: APOA1 (Figure 4F), a negative acute phase protein implicated in cholesterol transport and lipid metabolism, and A1AT, the most abundant circulating serine proteinase inhibitor, with a duplex immunomodulatory and anti-inflammatory function. Similarly, a deficiency of A1AT has been documented in patients with cluster headache [41], while it was increased in serum of MOH patients [16] and PM women [18]. The high presence of A1AT in serum samples could reflect and explain the low levels found in urine. Otherwise, TTHY (Figure 4G), a thyroid hormone-binding protein, was found significantly over-expressed in the same comparison. It has been hypothesized that sex hormones may up-regulate the TTHY expression at the mRNA level, which is followed by a concomitant and consistent rise of TTHY protein in the peripheral circulation [42]. TTHY was earlier found to also be increased in urine and serum of MOH patients [13,16], and in rats after sciatic nerve ligation [17]. In addition, peptides implicated in protein metabolic/catabolic processes (RS27A, PEPA3), production of choline acetyltransferase in neurons (PEBP1), and immunoglobulins involved in humoral immunity (IGHG2, KVD20, IGKC) were found to be increased in one or both migraineur groups.

Altogether, we essentially discovered dysregulated immune, metabolic, and, mainly, inflammation-related proteins. Although, to date, the exact etiology of MM and PM remains incompletely understood, an inflammatory and immune response, as well as factors associated with pain transmission, are generally accepted as possible mechanisms for the pathogenesis of migraine [43,44]. The present proteomic mass spectrometry-based research underlines significant changes in the urinary proteome of MM and PM groups with respect to controls, in accordance with our previous study conducted in serum samples [18]. Noteworthy, ITIH4, APOA1, A1AT, TTHY, and IGKC matched between the two studies. To strengthen the results, S10A8, UROM, AMBP, GELS, PTGDS, APOA1, and TTHY were further validated and quantified by immunoblot (Figure 4). Distinctively, this protein cluster might represent a selection of non-invasive biomarkers, to be considered for an advanced evaluation of these specific forms of migraine.

However, it is important to point out that the major limitation of this cross-sectional comparative proteomic research is the small sample size. Consequently, the present results can be considered as preliminary outcomes, which need to be validated in a wider set of samples. Moreover, urine collection was performed in fertile women during the follicular phase, before the ovulatory peak, but it would be interesting to also analyze urine from the luteal phase. Nonetheless, as far as we know, this is the first study providing information about the presence of differentially expressed urinary proteins in MM and PM. The proteomic workflow still requires complex performances, as well as expensive and time-consuming procedures, so currently, it can be just one of the initial steps in identifying novel candidate biomarkers of migraine. Once confirmed and validated in further analyses, these protein targets might be included in faster, cheaper, and high-throughput routine clinical tests.

## 5. Conclusions

Twenty-one urinary proteins were found dysregulated in MM and PM women, and 15 fell into one large protein-protein interaction network widely involved in inflammation. Thereby, we suppose that MM could be associated with a significant inflammatory response, only partially dependent on hormonal fluctuations. Indeed, the MM women showed an over-expression of urinary proteins indicative of a higher inflammatory condition with respect to the age-matched control group. In PM women, in which migraine persisted even after menopause, an increased expression of proteins implicated in metabolic processes and considered markers of CVD was found in addition to other proteins indicative of inflammation, immune, and anti-inflammatory responses activation.

In conclusion, the urinary proteomic approach can provide new insights into the mechanisms of MM and PM. The biological functions of the detected proteins and the great correspondence with our earlier outcomes on migraine might suggest their possible involvement in the pathophysiology of MM and PM. Hence, the identified protein cluster is worthy of further exploration; large-scale studies might contribute to validate these candidate biomarkers as distinctive and selective molecular targets for future clinical applications in migraine diagnosis and treatment.

## Figures and Tables

**Table 1:** Differentially expressed proteins detected by SDS-PAGE and identified by LC-MS/MS analysis.

Protein Full Name	Entry Name ^(a)^	Fold Change ^(b)^	*p*-Value ^(c)^
Uromodulin	UROM	+2.00 * +1.95 ^#^	0.013 0.011
Alpha-1-microglobulin Vesicular integral-membrane protein VIP36	${}_{\mathrm{LMAN}2}^{\mathrm{AMBP}}]$	+2.51 * +1.67 ^#^	0.001 0.005
Immunoglobulin kappa constant	IGKC	+1.58 *	0.013

^(a)^ Protein entry name from the UniProt database with extension Human. ^(b)^ Fold-change expressed as total band intensity increase in: * PM vs CTRL, ^#^ PM vs MM. ^(c)^ Statistically significant differences (*p* ˂ 0.05) among the groups. UROM (uromodulin), AMBP (alpha-1-microglobulin), LMAN2 (vesicular integral-membrane protein VIP36), IGKC (immunoglobulin kappa constant).

**Table 2:** Differentially expressed urinary proteins identified by LC-MS/MS after 2DE analysis.

Entry ^(a)^	Protein Full Name	Acc. ^(b)^	Gene ^(c)^	Score ^(d)^	Pep./Seq. ^(e)^	emPAI ^(^^f)^	Change ^(g)^	Function ^(h)^
S10A8	Protein S100-A8	P05109	S100A8	174	9/5	4.31	Up ^(1)^	Inflammation
KNG1	Kininogen-1	P01042	KNG1	314	12/10	0.68	Up ^(1)^	Inflammation
ALBU	Albumin	P02768	ALB	122	8/8	0.54	Up ^(1)^	Multifunction
IGHG2	Immunoglobulin heavy constant gamma 2	P01859	IGHG2	98	4/4	0.51	Up ^(1)^	Immune response
PEBP1	Phosphatidylethanolamine-binding protein1	P30086	PEBP1	50	5/5	1.45	Up ^(1)^	Neurotransmitter synthesis
ITIH4	Inter-alpha-trypsin inhibitor heavy chain H4 (fragment)	Q14624	ITIH4	125	6/4	0.16	Up ^(2)^	Inflammation
UROM	Uromodulin	P07911	UMOD	190	3/3	1.10	Up ^(2)^	Regulation
AMBP	Alpha-1-microglobulin	P02760	AMBP	818	41/10	2.10	Up ^(2)^	Inhibition
LMAN2	Vesicular integral-membrane protein VIP36	Q12907	LMAN2	260	16/8	1.10	Up ^(2)^	Transport
ZA2G	Zinc-alpha-2-glycoprotein	P25311	AZGP1	65	3/2	0.12	Up ^(2)^	Immune response
MASP2	Mannan-binding lectin serine protease 2	O00187	MASP2	192	11/5	0.28	Up ^(2)^	Inflammation modulation
GELS	Gelsolin	P06396	GSN	30	3/3	0.14	Up ^(2)^	Inflammation
PTGDS	Prostaglandin-H2 D-isomerase	P41222	PTGDS	466	20/6	3.06	Up ^(2)^	Inflammatory mediator
SAP3	Ganglioside GM2 activator	P17900	GM2A	162	10/6	1.84	Up ^(2)^	Lipid transport
IGKC	Immunoglobulin kappa constant	P01834	IGKC	228	10/4	2.41	Up ^(2)^	Immunity
KVD20	Immunoglobulin kappa variable 3D20	A0C4DH25	IGKV3D-20	122	4/3	1.39	Up ^(2)^	Immunity
RS27A	Ubiquitin-40S ribosomal protein S27a	P62979	RPS27A	104	8/4	1.24	Up ^(2)^	Metabolic process
APOA1	Apolipoprotein A-I	P02647	APOA1	50	3/3	0.45	Down ^(3)^	Lipid metabolism
A1AT	Alpha-1-antitrypsin	P01009	SERPINA1	400	19/13	2.16	Down ^(3)^	Anti-inflammatory
TTHY	Transthyretin	P02766	TTR	50	3/2	0.59	Up ^(3)^	Hormone binding
PEPA3	Pepsin A-3	PP0DJD8	PGA3	98	6/2	0.35	Up ^(3)^	Regulation

^(a)^ Entry name from UniProt knowledge database (www.uniprot.org (accessed on 21/01/2021)). ^(b)^ Primary accession number from UniProt. ^(c)^ Protein gene name from UniProt. ^(d)^ The highest score obtained with MASCOT search engine. ^(e)^ Number of significant peptides/significant matched sequences. ^(f)^ emPAI: Exponentially modified protein abundance index. ^(g)^ Direction of change: up- or down-regulation, ^(1)^ only in MM vs. both CTRL and PM, ^(2)^ only in PM vs both CTRL and MM, ^(3)^ in both MM and PM vs CTRL. ^(h)^ Main molecular function/ biological process.

## Data Availability

Data are contained within the article and in the Supplementary Materials.

## Supplementary Materials

The following is available online at https://www.mdpi.com/article/10.3390/jcm10091854/s1. Table S1: Proteomics in migraine: a comparison of the current literature reported in the manuscript, Table S2: Characteristics of the subjects (*n* = 15 for each group) as reported in our previous study, Table S3: Protein bands intensity signal detected by western blot analysis.

## Abbreviations

A1AT	Alpha-1-antitrypsin
ALBU	Albumin
AMBP	Alpha-1-microglobulin
APOA1	Apolipoprotein A-I
CVD	Cardiovascular disease
2DE	Two-dimensional gel electrophoresis
GELS	Gelsolin
IGHG2	Immunoglobulin heavy constant gamma 2
IGKC	Immunoglobulin kappa constant
ITIH4	Inter-alpha-trypsin inhibitor heavy chain H4
KNG1	Kininogen-1
KVD20	Immunoglobulin kappa variable 3D20
LC-MS/MS	Liquid chromatography/mass spectrometry
LMAN2	Vesicular integral-membrane protein VIP36
MASP2	Mannan-binding lectin serine protease 2
MM	Menstrually related migraine group
MOH	Medication overuse headache
PEBP1	Phosphatidylethanolamine-binding protein 1
PEPA3	Pepsin A-3
PM	Post-menopause migraine group
PTGDS	Prostaglandin-H2 D-isomerase
RS27A	Ubiquitin-40S ribosomal protein S27a
S10A8	Protein S100-A8
SAP3	Ganglioside GM2 activator
SDS-PAGE	Mono-dimensional gel electrophoresis
TTHY	Transthyretin
UROM	Uromodulin
ZA2G	Zinc-alpha-2-glycoprotein
