# Supplementary material for: Urinary Proteomics Reveals Promising Biomarkers in Menstrually Related and Post-Menopause Migraine
Source: J Clin Med. 2021 Apr 24;10(9):1854. doi: 10.3390/jcm10091854 (PMC8123166; doi:10.3390/jcm10091854)
Supplement: Supplementary file 1 [file jcm-10-01854-s001.zip › Supplementary Table S1.pdf]

Supplementary Table S1. Proteomics in migraine: a comparison of the current literature reported in the manuscript.

Migraine type	Sample type	Techniques(a)	Mass instrument	Number of proteins(b)	Ref.(c)
**MOH**	urine	SDS-PAGE	ESI-QToF-LC/MS	8*	Bellei et.al. [12]
	urine	2-DE	ESI-QToF-LC/MS	21*	Bellei et.al. [13]
	urine	_	SELDI-Tof-MS	18 peaks	Bellei et.al. [13]
	urine	Western-blot	—	4§	Bellei et.al. [14]
	urine	ELISA test	—	1#	Bellei et.al. [14]
**MOH**	serum	SDS-PAGE	LC-MS/MS-QO	30*	Pellesi et. al. [16]
	serum	2-DE	LC-MS/MS-QO	12*	Pellesi et. al. [16]
	serum	ELISA test	—	4#	Pellesi et. al. [15]
**MM and PM**	serum	2-DE	LC-ESI-QTof-MS/MS + LC-ESI-QO-MS/MS	13*	Bellei et.al. [18]

MOH: Medication-overuse headache
MM: Menstrually-related migraine
PM: Post-menopause migraine

(a)Techniques: proteomic technique used in the referenced study.
(b)Number of proteins: total number of differentially expressed proteins identified by MS*, or validated§ and quantified# by complementary proteomic methods.
(c)Ref.: reference number, as reported in the present manuscript.
