# Supplementary material for: Urinary Proteomics Reveals Promising Biomarkers in Menstrually Related and Post-Menopause Migraine
Source: J Clin Med. 2021 Apr 24;10(9):1854. doi: 10.3390/jcm10091854 (PMC8123166; doi:10.3390/jcm10091854)
Supplement: Supplementary file 1 [file jcm-10-01854-s001.zip › Supplementary Table S2.pdf]

Supplementary Table S2: Characteristics of the subjects (n=15 for each group), as reported in our previous study [18].

Variable	MM<br>n (%)	PM<br>n (%)	CTRL<br>n (%)
Mean age ± SD (years)	34.8±7.1	57.0±5.0[a]	29.2±7.2
Age range (years)	21÷45	51÷65	22÷45
BMI ± SD	23.1±2.5	24.1±4.1	23.3±4.2
Smoker	2 (13)	1 (7)	3 (20)
Years of migraine ± SD	16.8±8.5	33.1±13.5[b]	0 (0)
Migraine days/3 months ± SD	25.7±17.2	27.3±19.2	0 (0)
**Acute migraine treatment**			
Triptans	9 (60)	8 (53)	0 (0)
NSAIDs	8 (53)	9 (60)	0 (0)
Analgesic combination	2 (13)	2 (13)	0 (0)
**Comorbidity**			
Hypertension	1 (7)	4 (27)	1 (7)
Allergic rhinitis	3 (20)	3 (20)	4 (27)
Hypercholosterolemia	1 (7)	3 (20)	0 (0)
Thyroiditis	1 (7)	4 (27)	1 (7)
**Concomitant treatment**			
Antihypertensive agents	1 (7)	4 (27)	1 (7)
Antihistamines	3 (20)	3 (20)	4 (27)
Statins	1 (7)	3 (20)	0 (0)
Levothyroxine	1 (7)	4 (27)	1 (7)

[a] PM *vs* MM group, P<0.01; PM *vs* CTRL group, P<0.001; one-way ANOVA test

[b] PM *vs* MM group, P<0.001; one-way ANOVA test
