# Supplementary material for: Urinary Proteomics Reveals Promising Biomarkers in Menstrually Related and Post-Menopause Migraine
Source: J Clin Med. 2021 Apr 24;10(9):1854. doi: 10.3390/jcm10091854 (PMC8123166; doi:10.3390/jcm10091854)
Supplement: Supplementary file 1 [file jcm-10-01854-s001.zip › Supplementary Table S3 .pdf]

Supplementary Table S3. Protein bands intensity signal obtained by Western-blot analysis.

	CTRL group	MM group	PM group	p-value
**APOA1**	1358 ± 116	139 ± 52	78 ± 44	0.00008 (MM vs CTRL)<br>0.00006 (PM vs CTRL)
**TTHY**	886 ± 191	4149 ± 1112	3685 ± 1028	0.0074 (MM vs CTRL)<br>0.0097 (PM vs CTRL)
**S10A8**	697 ± 233	1917 ± 151	1304 ± 330	0.0016 (MM vs CTRL)<br>0.0429 (MM vs PM)
**UROM**	717 ± 64	893 ± 100	1170 ± 99	0.0027 (PM vs CTRL)<br>0.0268 (PM vs MM)
**AMBP**	404 ± 157	836 ± 237	1694 ± 92	0.00025 (PM vs CTRL)<br>0.00426 (PM vs MM)
**GELS**	592 ± 75	727 ± 159	1514 ± 172	0.0011 (PM vs CTRL)<br>0.0044 (PM vs MM)
**PTGDS**	4485 ± 1235	5570 ± 1216	9163 ± 1183	0.0091 (PM vs CTRL)<br>0.0214 (PM vs MM)

Reported data are indicative of protein intensity signal derived from band optical density and area detected in each group. Data are expressed as mean of three independent experiments for each protein ± standard deviation. P-values derived from Student's *t*-test ($p<0.05$ is considered as statistically significant).
